# Benign Ovarian Tumors in Pregnancy: A Case Report of Metachronous Ipsilateral Recurrent Mucinous Cystadenoma in Initial Pregnancy and Mature Cystic Teratoma in Subsequent Pregnancy

**DOI:** 10.7759/cureus.3818

**Published:** 2019-01-03

**Authors:** Arielle M Schreck, Hana F Mikdachi

**Affiliations:** 1 Obstetrics and Gynecology, East Tennessee State University, Quillen College of Medicine, Johnson City, USA

**Keywords:** adnexal mass in pregnancy, metachronous tumor, synchronous tumor, mucinous cystadenoma, benign mature teratoma

## Abstract

Mucinous cystadenomas of the ovary are benign epithelial neoplasms that can grow rapidly during pregnancy. They may cause ovarian torsion, virilization, inferior vena cava syndrome, and even preterm labor and fetal growth restriction. Various theories exist regarding the pathogenesis of these tumors. One hypothesis suggests that they may arise from teratomas. Our case report describes synchronous mucinous cystadenomas and ovarian teratomas, as well as metachronous mucinous cystadenomas in patients with a history of ovarian teratoma. There has been no report of metachronous ipsilateral teratoma after previous mucinous cystadenoma. We present a 22-year-old female with a history of bilateral ovarian tumors in a prior pregnancy noted to have a recurrent ovarian mass on her left ovary at the time of cesarean section of a subsequent pregnancy. She had two prior cystectomies for metachronous mucinous cystadenomas of her left ovary, and a right salpingo-oophorectomy for the ovarian torsion in her previous pregnancy. On her current pregnancy, she developed a mature cystic teratoma of the remaining left ovary. The rapid growth and recurrence rate of these tumors highlights the importance of close surveillance of ovarian masses during pregnancy, even those that seem benign. In this case, a history of unilateral salpingo-oophorectomy with multiple contralateral cystectomies did not appear to affect her fertility. Her future ovarian reserve is unknown, pointing to the need for adequate pre-operative counseling in similar cases of ovarian masses in pregnancy.

## Introduction

The majority of adnexal masses in pregnancy resolve spontaneously [1]. Those that persist may rupture, infarct, or cause ovarian torsion. Other complications include fetal growth restriction and preterm labor. The most common benign ovarian neoplasms are serous or mucinous cystadenomas and mature cystic teratomas. In pregnancy, mucinous cystadenomas may grow so large that they result in persistent supine hypotensive syndrome [2]. Case reports have described these tumors reaching up to 30 to 40 centimeters in diameter during pregnancy [2-3]. 

## Case presentation

Presenting concerns

This is a case of a 22-year-old white female who presented at 34 weeks with preterm premature rupture of membranes. She had a history of two prior cesarean deliveries. A left ovarian tumor, not noted during the course of her current pregnancy, was noted on cesarean delivery.

Current pregnancy

**Figure 1:**
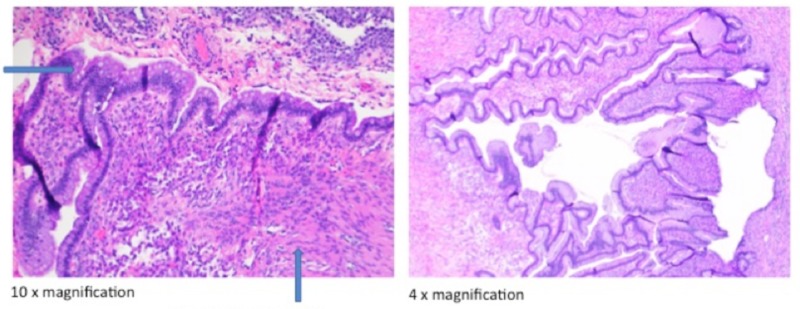
Mature cystic teratoma The arrow pointing to the right identifies endodermal elements. The arrow pointing up identifies mesodermal elements.

When she presented with preterm premature rupture of membranes, repeat cesarean section was performed again. At the time of surgery, another 8 cm mass on her left ovary was diagnosed intraoperatively. This mass was not diagnosed prior to surgery. A second trimester ultrasound was performed that did not show any ovarian cyst. During the surgery, the mass appeared tan with punctate focal hemorrhage. The mass was unable to be separated from her left fallopian tube, so the mass, the left fallopian tube and part of the left ovary were all removed. We were able to leave a small amount of residual ovarian tissue. Pathologic description noted a multiloculated and cystic mass with clear mucinous fluid consistent with mature cystic teratoma (Figure 1). Her postoperative course was uncomplicated.

Previous pregnancy

**Figure 2:**
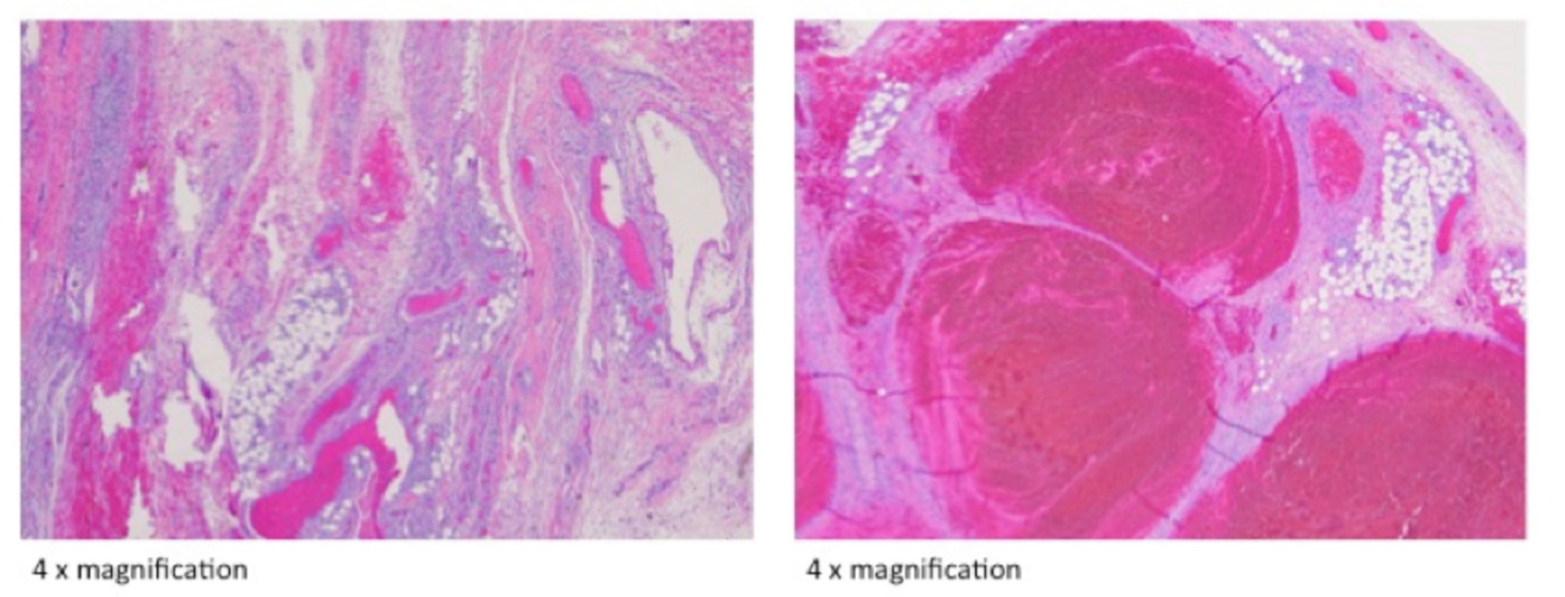
Ovarian torsion No viable ovarian stroma can be identified.

**Figure 3:**
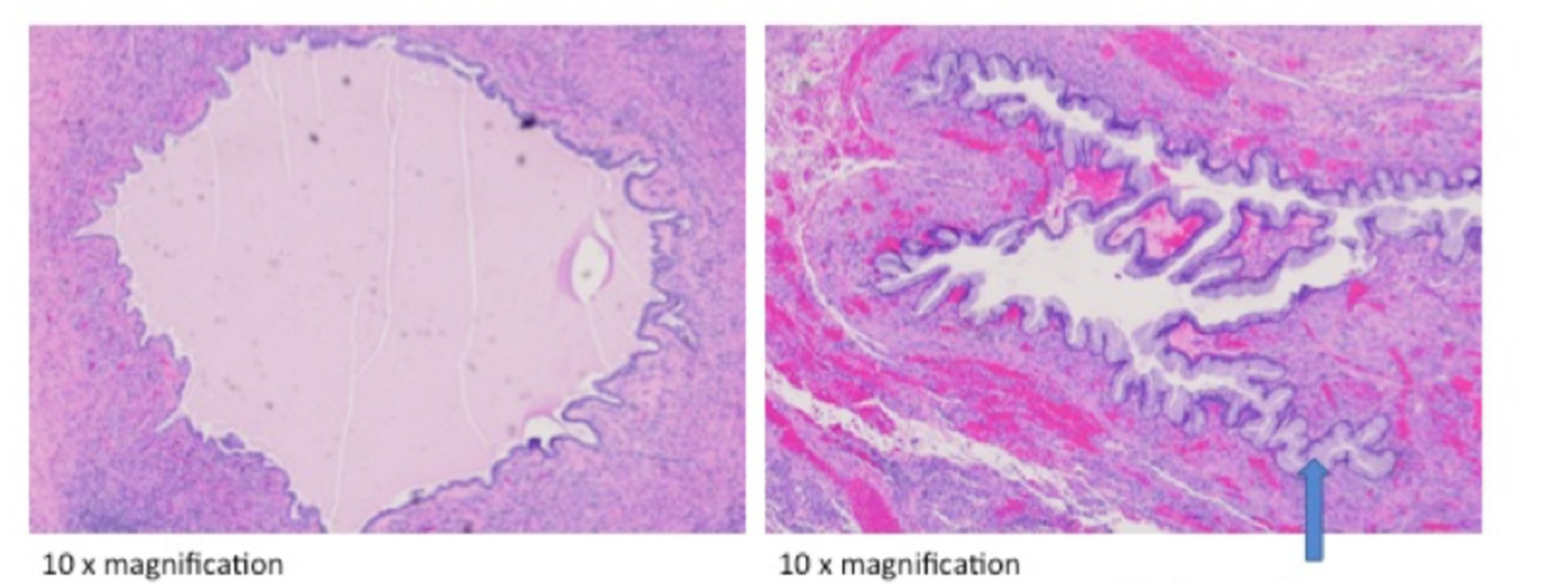
Mucinous cystadenoma Mucinous columnar epithelium lines the cyst walls.

Her antecedent pregnancy was complicated by right ovarian torsion at 13 weeks secondary to an ovarian tumor. A right salpingo-oophorectomy was performed. The right ovary was sent to pathology and noted to be infarcted with no viable tissue from the mass to make a definitive diagnosis (Figure 2). During that same surgery, an 8 cm, irregularly shaped mass with a focally hemorrhagic surface was noted on the left ovary. A left ovarian cystectomy was performed, and final pathology showed a benign mucinous cystadenoma (Figure 3). At the time of cesarean in the same pregnancy, a metachronous mucinous cystadenoma was noted on her left ovary; cystectomy and partial left oophorectomy were performed.

Follow-up and outcomes

Following her third cesarean delivery, this patient was surprised to learn that she had developed another ovarian cyst in pregnancy necessitating removal. She was concerned about the function of her residual ovarian tissue following three surgeries. 

## Discussion

This patient likely had accelerated growth of metachronous tumors during her antecedent and current pregnancy. It is well known that mucinous cystadenomas may grow rapidly during pregnancy by an unknown mechanism. Some theories suggest that these tumors may be positive for estrogen, progesterone, and human chorionic gonadotropin (HCG) receptors; however, there have been cases of receptor-negative tumors that expand rapidly during pregnancy as well [2].

In this case, both the patient and her physicians were unaware that she had another ovarian mass until the time of cesarean. One large retrospective study demonstrated that the majority of adnexal masses in pregnancy may be incidentally discovered at the time of cesarean delivery (0.3% of all cesarean deliveries), with dermoid cysts being the most common diagnosis [4].

Other case studies have reported that rapid growth of teratomas during pregnancy recur during subsequent pregnancies, even on the contralateral ovary [5]. One of the risk factors is a history of prior surgeries, including the cesarean deliveries in our case [5]. This case is unique in that the tumor recurred during two consecutive pregnancies. It is unknown if the mature cystic teratoma developed by chance, by unknown cytogenetic factors, or in relation to the mucinous cystadenomas.

The pathogenesis of mucinous cystadenomas is unknown. Studies have distinguished mucinous tumors of primary ovarian mucinous origin from metastases of the lower intestinal tract using tumor markers. Primary ovarian mucinous tumors express cytokeratin 7 (CK7) and variably express cytokeratin 20 (CK20); whereas, tumors that do not express CK7 but express CK20 likely arose from the gastrointestinal tract [6]. One case report has used these biomarkers to demonstrate how a mucinous cystadenoma originated from the colonic epithelium of a mature cystic teratoma, because the mature cystic teratoma demonstrated features of both the classic mucinous cystadenoma and a normal colonic wall [7].

The synchronous occurrence of mucinous cystadenoma and mature teratomas is well documented in the literature [8-10]. Some authors suggest that mucinous cystadenomas arise from the totipotent cells within a teratoma [8]. In 2010, Parmentier et al. described a rare case of metachronous mature teratoma and mucinous cystadenoma in an adolescent, with the antecedent tumor being a teratoma. He subsequently develops three hypotheses: 1) these two tumors occurred by chance alone, 2) these tumors occurred in a tumor-susceptible ovary, and 3) the antecedent teratoma recurred as a mucinous cystadenoma [9].

This last hypothesis is referring to the coexistence of two tumors within one tumor, also cited as a “collision tumor” by other authors [11]. One case report described the growth of a mature teratoma within a large mucinous cystadenoma, and attributed the development of the teratoma to either epithelial metaplasia of the lining of the cystadenoma or from cell rests within the neoplasm [12].

This case demonstrates yet another example in which mucinous cystadenomas and mature teratomas seemed to co-occur. However, the cause and effect relationship between these two tumors is unknown.

## Conclusions

This report presents a case in which pregnancies accelerate the growth of both unilateral and bilateral ovarian masses. These tumors may potentially lead to complications that have been cited in the literature, including ovarian torsion, intrauterine growth restriction, and preterm deliveries. Women with a history of ovarian cysts or tumors should be counseled during pregnancy about a possible recurrence, and careful ultrasound surveillance should be undertaken. Despite multiple ovarian surgeries, this patient was quickly able to achieve pregnancy. Any woman with a history of ovarian tumors should be counseled prior to surgery about this risk of recurrence and the variable fertility outcomes that may follow.
